# Phase Prediction of High-Entropy Alloys by Integrating Criterion and Machine Learning Recommendation Method

**DOI:** 10.3390/ma15093321

**Published:** 2022-05-05

**Authors:** Shuai Hou, Yujiao Li, Meijuan Bai, Mengyue Sun, Weiwei Liu, Chao Wang, Halil Tetik, Dong Lin

**Affiliations:** 1 School of Information and Electrical Engineering, Hebei University of Engineering, Handan 056038, China; houshuai20072@163.com (S.H.); liyujiao2495@163.com (Y.L.); 18749309173@163.com (M.S.); wangchao2019@hebeu.edu.cn (C.W.); 2 School of Mechanical Engineering, Dalian University of Technology, Dalian 116024, China; liuww@dlut.edu.cn; 3 Industrial and Manufacturing Systems Engineering, Kansas State University, Manhattan, KS 66506, USA; haliltetik@ksu.edu

**Keywords:** high-entropy alloys, phase prediction, machine learning recommendation, criterion

## Abstract

The comprehensive properties of high-entropy alloys (HEAs) are highly-dependent on their phases. Although a large number of machine learning (ML) algorithms has been successfully applied to the phase prediction of HEAs, the accuracies among different ML algorithms based on the same dataset vary significantly. Therefore, selection of an efficient ML algorithm would significantly reduce the number and cost of the experiments. In this work, phase prediction of HEAs (PPH) is proposed by integrating criterion and machine learning recommendation method (MLRM). First, a meta-knowledge table based on characteristics of HEAs and performance of candidate algorithms is established, and meta-learning based on the meta-knowledge table is adopted to recommend an algorithm with desirable accuracy. Secondly, an MLRM based on improved meta-learning is engineered to recommend a more desirable algorithm for phase prediction. Finally, considering poor interpretability and generalization of single ML algorithms, a PPH combining the advantages of MLRM and criterion is proposed to improve the accuracy of phase prediction. The PPH is validated by 902 samples from 12 datasets, including 405 quinary HEAs, 359 senary HEAs, and 138 septenary HEAs. The experimental results shows that the PPH achieves performance than the traditional meta-learning method. The average prediction accuracy of PPH in all, quinary, senary, and septenary HEAs is 91.6%, 94.3%, 93.1%, and 95.8%, respectively.

## 1. Introduction

High-entropy alloys are composed of multiple (not less than five) main elements [1,2]. They typically possess high hardness, high strength, high temperature-softening resistance, superior wear resistance, and corrosion resistance [3]. HEAs have broad application prospects in the nuclear power industry, biochemistry, chemical industry, etc. [4]. The phases of HEAs mainly include solid solution (SS), intermetallic compound (IM), solid solution and intermetallic compound (SS+IM), and amorphous phase (AM) [5,6,7]. Because these phases are key factors determining the performance of materials, the accurate prediction of the phases in HEAs is crucial for material design [8].

In recent decades, many phase prediction methods for HEAs have been applied in the materials field. However, the quantity of element combination of HEAs is much larger than single-principal component alloys, so it is more difficult to predict the phases of HEAs. The traditional trial-and-error method is an approach to detect the phases of HEAs that has low efficiency, long cycle time, and high cost [9]. The functional density theory and calculation of phase diagram method (CALPHAD) are other methods to predict the phases of HEAs; both are inefficient and have a heavy computational burden [10]. To address these issues, parameter methods based on criterion have been researched. Yang [5] proposed the parameter Ω and pointed out that SS was easily formed when Ω≥1.1 and δ≤6.6%, where δ is the is the mean square deviation of the atomic size of all elements in multi-principal alloys. Similarly, Zhang et al. [11] confirmed that SS was easily formed if Ω≥1.1 and δ≤6.6%. Guo [12] concluded that ∆*H_mix_* and ∆*S_mix_* can determine SS, where ∆*H_mix_* is the enthalpy of mixing and ∆*S_mix_* is the entropy of mixing. Tan [13] demonstrated that the SS was formed under the same conditions in the literature [5]. However, the above-mentioned parameter methods have limited application space outside these criteria. In addition, the establishment process of these criteria requires heavy workload and time consumption implications.

Some scholars have predicted the phases of HEAs by ML algorithms. These ML algorithms can establish the mapping relationship between the input parameters and phases of HEAs with extensive training [14,15,16,17,18]. Islam et al. [19] classified the phases of HEAs as SS, IM, and AM by multi-layer neural network algorithm. Huang et al. [20] predicted the phases of HEAs by K-nearest neighbor (KNN), support vector machine (SVM), and artificial neural network (ANN). Qu et al. [21] established a universal method by SVM to predict the phases of HEAs. Li et al. [22] adopted SVM to distinguish the phases of HEAs based on cross validation method, which achieved better accuracy than CALPHAD. Although many ML algorithms have been applied to predict the phases of HEAs and accomplished desirable results, the accuracies of different ML algorithms based on the same dataset vary considerably. The famous theorem free lunch (NoFreeLunch, NFL) in the machine learning field [23] shows that there is no ‘general algorithm’ that can solve all problems once and for all. If material designers can select the ML algorithm with the most desirable accuracy, it will significantly reduce the number of experiments, accrued time, and cost savings. The issue of algorithm selection is still challenging and time-consuming for material designers.

Therefore, the selection of an appropriate algorithm has attracted great interest. Khan et al. [24] reviewed the meta-learning algorithm, which can recommend a desirable algorithm. Aguiar et al. [25] adopted meta-learning to select the most suitable image segmentation ML algorithm and obtained desirable results. Chu et al. [26] proposed an adaptive recommendation model based on meta-learning that maps the relationship between the performance of algorithms and datasets so as to recommend ideal algorithms in different datasets. Cui [27] proposed a general meta-modeling recommendation system based on meta-learning that can automatically recommend desirable algorithms for researchers. The meta-learner is a key component that considerably affects the accuracy of meta-learning. Pimentel et al. [28] adopted KNN as the meta-learner in meta-learning to recommend a suitable ML algorithm for a new dataset. Ferrari et al. [29] adopted KNN to recommend algorithms in meta-learning and achieved good results. However, when there are noise points and the useful neighbor information of each sample is not considered, the performance of KNN is poor. Song et al. [30] proposed a decremental instance selection for KNN regression (DISKR) and achieved better experimental results than with KNN alone. Zhang [31] designed a shelly nearest neighbor (SNN) algorithm that considers the information of the left and right nearest neighbors of the test sample and obtains a better result than KNN. Although the single data-driven model has achieved success in several fields, it has poor interpretability and generalization and requires a large number of experimental samples. Additionally, literature about meta-learning in the phase prediction of HEAs is still scarce.

Some scholars have carried out research combining physical models and data-driven models. These models benefit from the advantage of the powerful learning ability of data-driven models and the strong interpretability and generalization of physical models. Such models have achieved desirable results in a wide range of areas. Lv et al. [32] proposed a novel prediction model constituted by the mechanism method and ML model to forecast the liquid steel temperature in a ladle furnace. Later on, Lv et al. [33] proposed a novel steel temperature prediction model based on an ML algorithm and first-principle method. Hou et al. [34] proposed a framework composed of the hard division model and prediction model that combines the mechanism model and ML model.

However, the literature rarely addresses how to recommend an ideal algorithm for HEAs. In this paper, a PPH constituted by MLRM and criterion of SS is proposed to provide desirable results for material designers. First, a meta-knowledge table with meta-features of datasets and accuracies of algorithms is established, and meta-learning is adopted to recommend an ideal algorithm for material designers. Secondly, an MLRM based on improved meta-learning is proposed to recommend a more desirable algorithm. Finally, the PPH based on criterion of SS and the MLRM is proposed to predict the phases of HEAs. Compared with other ML algorithms in the HEA field, the method proposed in this paper can recommend an ML algorithm with ideal accuracy for material designers, which can reduce the burden on material designers in selecting algorithms, effectively improve the prediction accuracy of phases, and greatly reduce the experimental time and cost.

## 2. Preliminaries

### 2.1. Research Background of HEAs

HEAs have excellent properties that are considerably affected by their phase structures [35]. The phases of HEAs include SS, IM, SS+IM, and AM. Some scholars have adopted the parameter method to determine the phase formation of HEAs in the materials field [5,11,12,13].

To date, the main parameters of HEAs include δ, $\Delta H_{mix}$, Ω, Δχ, and $\Delta S_{mix}$. The formulae of these parameters are shown in Equations (1)–(5) [11,12]:

(1) $$\delta =\sqrt{\sum_{i=1}^{n}c_{i}{\left(1-r_{i}/\bar{r}\right)}_{}^{2}}$$

where δ is the mean square deviation of atomic size of all elements in multi-principal alloy, *c_i_* is the atomic percentage of the *i*-th element, *r_i_* is the atomic radius of the *i*-th principal element, and $\bar{r}$ is the weighted average atomic radius of all principals, $\bar{r}=\sum_{i=1}^{n}c_{i}r_{i}$.

(2) $$\Delta H_{mix}=\sum_{i=1,i<j}^{n}4H_{ij}c_{i}c_{j}$$

where $\Delta H_{mix}$ is the enthalpy of mixing; *H_ij_* is the mixing enthalpy of *i*-th and *j*-th binary liquid alloy; and *c_i_* and *c_j_* are the atomic percentage of the *i*-th and *j*-th element, respectively.

(3) $$\Delta S_{mix}=-R\sum_{i=1}^{n}c_{i}\ln c_{i}$$

where $\Delta S_{mix}$ is the entropy of mixing; *c_i_* is the atomic percentage of the *i*-th element; and *R* is the gas constant, the value of which is 8.314 J·K^−1^·mol^−1^.

(4) $$\Omega =\frac{T_{m}\Delta S_{mix}}{\left|\Delta H_{mix}\right|}$$

where Ω is the number of states of multi-principal element alloy system molecules; and *T_m_* is the weighted average melting point for all elements, $T_{m}=\sum_{i=1}^{n}c_{i}T_{m}^{i}$.

(5) $$\Delta \chi =\sum_{i=1}^{n}c_{i}(\chi_{i}-\bar{\chi })$$

where Δχ is the electronegativity difference of the HEA system, and $\bar{\chi }=\sum_{i=1}^{n}c_{i}\chi_{i}$, $\chi_{i}$ is the electronegativity of constituent elements.

### 2.2. Meta-Learning

**Figure 1:**
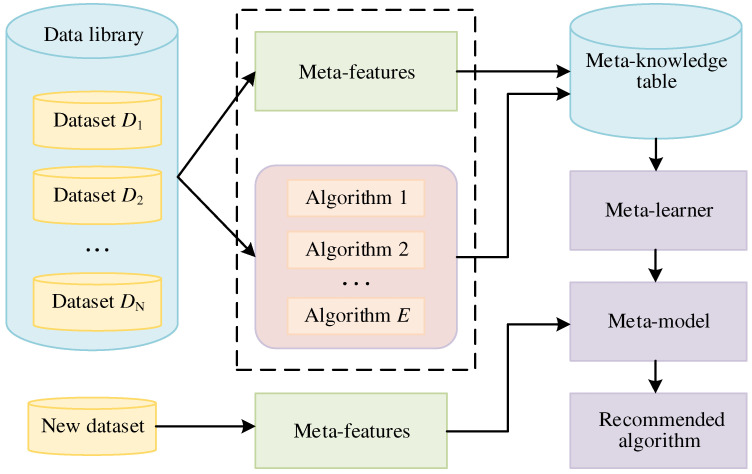
Schematic diagram of meta-learning.

Meta-learning is utilized to solve the problem of algorithm selection [36,37]. The meta-learning method establishes the relationship between different datasets and the performance of algorithms to recommend the most appropriate algorithm. A schematic diagram of meta-learning is shown in Figure 1.

In Figure 1, the data library contains *N* datasets, including *D*_1_, *D*_2_, …, *D*_N_. The selection of meta-features significantly affects the performance of meta-learning. The simple, statistical, and information theoretic meta-features can reflect the characteristic of several datasets [24,26,29,38]. In Figure 1, there are *E* candidate algorithms. The meta-knowledge table is constructed by meta-features and performance of candidate algorithms. The meta-learner is a very important part of meta-learning. The meta-learner is trained based on the meta-knowledge table, which takes the meta-features of datasets as input variables and yields the performance of algorithms as output variables. The KNN method is often used as a meta-learner in the literature [28,29]. The meta-model is a trained meta-learner that maps relationships between the meta-features of datasets and the performance of candidate algorithms. The algorithm steps for meta-learning are as follows:

Step 1: Compute the meta-features of each dataset.

Step 2: Train each algorithm on each dataset to evaluate its performance.

Step 3: Construct the meta-knowledge table with the meta-features of datasets and the performance of candidate algorithms.

Step 4: Train the meta-learner based on the meta-knowledge table to map the relationship between meta-features and performance of the candidate algorithms. The trained meta-learner is also called the meta-model.

Step 5: Compute the meta-features of the new dataset and predict the performance of candidate algorithms on the new dataset by the meta-model. Select the algorithm with the highest predictive performance as the recommended algorithm.

### 2.3. Shelly Nearest Neighbor

The SNN is a neighbor-instance selection method for classification and regression problems. Given an instance, its shelly nearest neighbors refer to the nearest neighbors that make up the shell to encapsulate the instance [39]. The SNN method considers its left and right nearest neighbors of each attribute in a given dataset [31]. The specific steps of the SNN algorithm are as follows [40].

Let $F=\left\{f_{1},f_{2},\ldots ,f_{R}\right\}$ be the set of *R* class labels and $D=\{(x_{1},y_{1}),(x_{2},y_{2}),\ldots ,(x_{N},y_{N})\}$ be the dataset consisting of *N* instances, where *x_i_* is a vector of *M* attributes, and $y_{i}\subseteq F$ is the label of the *i*-th instance, *x_i_*.

For the *j*-th attribute (1≤j≤M), the left nearest neighbor of a query instance, *x_t_*, within *D* refers to the instances whose value on the *j*-th attribute is smaller than *x_t_* but larger than the rest. The left nearest neighbor of a query instance, *x_t_*, on the *j*-th attribute is defined as follows (Equation (6)):

(6) $$x_{t}^{-}(D,j)=\{x_{i}\in D|x_{kj}\leq x_{ij}\leq x_{tj},1\leq i,k\leq N\}$$

where *x_ij_* is the *j*-th attribute value of *x_i_*. According to this definition, if *x_tj_* was not the smallest one, *x_t_* has at least one left nearest neighbor, $x_{i}\in D$, on the *j*-th attribute, such that $x_{ij}\leq x_{tj}$, whereas $x_{kj}\leq x_{ij}$ for the remaining instances, *x_k_*, within *D*. Based on Equation (6), the left nearest neighbors of *x_t_* over all attributes within *D* are represented as Equation (7):

(7) $$x_{t}^{-}(D)=\cup_{j=1..M}x_{t}^{-}(D,j)$$

In a similar way, the right nearest neighbors of *x_t_* over all attributes within *D* are represented as Equation (8):

(8) $$x_{t}^{+}(D)=\cup_{j=1..M}x_{t}^{+}(D,j)$$

where $x_{t}^{+}(D,j)$ is the right nearest neighbors of *x_t_* with respect to the *j*-th attribute, as shown in Equation (9):

(9) $$x_{t}^{+}(D,j)=\{x_{i}\in D|x_{kj}\geq x_{ij}\geq x_{tj},1\leq i,k\leq N\}$$

The SNN of *x_t_* within *D* refers to its left and right nearest neighbors, as shown in Equation (10):

(10) $$SNN(x_{t})=x_{t}^{+}(D)\cup x_{t}^{-}(D)$$

**Figure 2:**
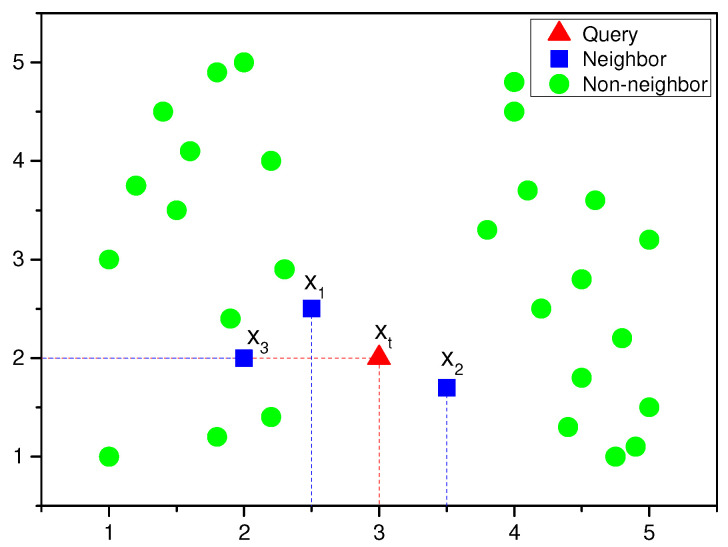
SNN of the query instance.

Generally speaking, there are about 2×M shelly nearest neighbors for *x_t_* if *D* has *M* attributes. For ease of understanding, the SNN of the query instance is shown in Figure 2.

Figure 2 shows an instances diagram of two-dimensional distribution. The red triangle is the query instance, marked as Query. The blue quadrangle is the neighbor instances of the query instance, which are selected by the SNN. *x_t_* is the query instance. The right nearest neighbor set, $x_{t}^{+}(D,j)$, is composed of all instances in dataset *D* whose values are greater than or equal to *x_tj_*. The left nearest neighbor set, $x_{t}^{-}(D,j)$, is composed of all instances in dataset *D* whose values are less than or equal to *x_tj_*. The total dimension number is *M* = 2, where *j* = 1 represents the horizontal axis variable and *j* = 2 represents the vertical axis variable. *x_t_*_1_ and *x_t_*_2_ represents the horizontal and vertical axis value of the query instance, respectively.

The selection process of neighbor instances of *x_t_* is as follows: First, the horizontal axis value and the vertical axis value of all instances in the dataset, *D*, are compared with *x_t_*_1_ and *x_t_*_2_, respectively. Secondly, if the value of the horizontal axis variable of the instances is greater or less than *x_t_*_1_, these instances form the instance set $x_{t}^{+}(D,1)$ or $x_{t}^{-}(D,1)$. If the value of the vertical axis variable of the instances is greater or less than *x_t_*_2_, these instances form the instance set $x_{t}^{+}(D,2)$ or $x_{t}^{-}(D,2)$. Thirdly, the left and right nearest neighbor of query instance *x_t_* along the horizontal axis are *x*_1_ and *x*_2_, which are the maximum or minimum in $x_{t}^{-}(D,1)$ or $x_{t}^{+}(D,1)$. Fourthly, the instance with a maximum value in $x_{t}^{-}(D,2)$ is *x*_3_, and the instance with a minimum value in $x_{t}^{+}(D,2)$ is the same instance, *x*_3_. The left and right nearest neighbor of query instance *x_t_* along the vertical axis is the same instance, *x*_3_. Thus, the shelly nearest neighbor set of the query sample *x_t_* instance includes instances *x*_1_, *x*_2_, and *x*_3_.

### 2.4. Decremental Instance Selection for KNN Regression

Decremental instance selection for KNN regression was first proposed by Song [30]. DISKR is an effective instance selection algorithm for KNN regression that removes outliers and the samples with less effect in the training set for KNN.

The KNN regressor is learned by comparing the given test instances with the training set. Let $D=\{(x_{1},y_{1}),(x_{2},y_{2}),\ldots ,(x_{N},y_{N})\}$ be the training set, where $x_{i}=(x_{i1},x_{i2},\ldots ,x_{iM})$ is the *i*-th instance with *M* attributes, and *y_i_* is the output of *x_i_*. *N* is the number of instances. When a query instance, *x_t_*, is given, the distance, *d_i_*, between *x_t_* and each instance, *x_i_*, in *D* is calculated first, and then *d_i_* is sorted by ascending order. The first *k* instances whose *d_i_* ranks ahead are selected as *k* nearest neighbors of *x_t_*, and the predicted output, $\hat{y_{t}}$, of *x_t_* is the average value of *y_i_* of *k* nearest neighbors.

The specific steps of DISKR are as follows:

Input: Dataset $D=\{(x_{1},y_{1}),(x_{2},y_{2}),\ldots ,(x_{N},y_{N})\}$, The parameter θ.

Output: The subset S⊆D.

Step 1: Remove outliers. If $PD(x_{i})=|y_{i}-\hat{y_{i}}|>(1-\theta )y_{i}$, then the instance *x_i_* is an outlier instance and removed; otherwise, it is not an outlier instance and is retained. *y_i_* is the output of *x_i_*; $\hat{y_{i}}$ is the predicted output of *x_i_*.

Step 2: Sort the remaining instances after removing outliers. Sort instances, *x_i_*, by the absolute difference, $PD(x_{i})=|y_{i}-\hat{y_{i}}|$, in descending order.

Step 3: Delete instances with less effect on the KNN regressor. The effect of *x_i_* could be estimated by the change in performance of KNN over *D* and $D-\{x_{i}\}$. The training error is used to approximately estimate the performance of KNN, which is expressed by the residual sum of squares (RSS).

$R_{bf}(x_{i})$ is the RSS on the *D*. $R_{af}(x_{i})$ is the RSS on the $D-\{x_{i}\}$, which represents the training set without *x_i_*. $R_{bf}(x_{i})$ and $R_{af}(x_{i})$ are shown in Equations (11) and (12):

(11) $$R_{bf}(x_{i})=\sum_{x_{j}\in D-\{x_{i}\}}{\left(y_{j}-\hat{y_{j}}\right)}^{2}$$

(12) $$R_{af}(x_{i})=\sum_{x_{j}\in D-\{x_{i}\}}{\left(y_{j}-\hat{{y^{\prime }}_{j}}\right)}^{2}$$

where $\hat{y_{j}}$ (1≤j≤N,j≠i) and $\hat{{y^{\prime }}_{j}}$ (1≤j≤N,j≠i) are the predicted output of KNN based on *D* and $D-\{x_{i}\}$.

The effect of *x_i_* on KNN is represented as Equation (13):

(13) $$\nabla (x_{i})=R_{af}(x_{i})-R_{bf}(x_{i})$$

After an instance, *x_i_*, is removed, the following rule is adopted to avoid a significant negative change in the performance of the regressor, as shown in Equation (14):

(14) $$\nabla (x_{i})\leq \theta R_{bf}(x_{i})$$

where θ∈(0,1) is the significant coefficient.

Step 4: Output the subset *S*; the remaining samples in the subset *S* are more relative samples that remove outliers and points that have less effect on KNN.

## 3. Methodology

In this section, the phase prediction of high-entropy alloys (PPH) is proposed by integrating machine learning recommendation method and criterion to predict the phases of HEAs.

### 3.1. Machine Learning Recommendation Method

**Figure 3:**
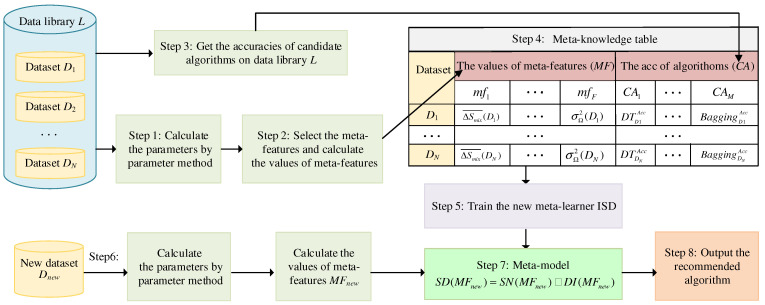
Schematic diagram of MLRM.

An MLRM based on improved meta-learning is proposed. The schematic diagram shown in Figure 3 illustrates MLRM, which can guide material designers to recommend an ideal algorithm.

As shown in Figure 3, the data library, $L=\{D_{1},D_{2},\ldots ,D_{N}\}$, contains *N* datasets. *D_i_* is the *i*-th dataset of *L*. The parameters $\Delta S_{mix}$, $\Delta H_{mix}$, δ, Δ*χ*, and Ω of each sample in each dataset, *D_i_*, are calculated by the parameter method [11,12]. The meta-features should reflect the characteristic of the datasets. The meta-feature set, $MF=\{mf_{1},mf_{2},\ldots ,mf_{F}\}$, includes $\bar{\Delta S_{mix}}$, $\bar{\Delta H_{mix}}$, $\bar{\delta }$, $\bar{\Delta \chi }$, $\bar{\Omega }$, $\sigma_{\Delta S_{mix}}^{2}$, $\sigma_{\Delta H_{mix}}^{2}$, $\sigma_{\delta }^{2}$, $\sigma_{\Delta \chi }^{2}$, and $\sigma_{\Omega }^{2}$. $\bar{\Delta S_{mix}}$, $\bar{\Delta H_{mix}}$, $\bar{\delta }$, $\bar{\Delta \chi }$, and $\bar{\Omega }$ are the mean value of $\Delta S_{mix}$, $\Delta H_{mix}$, δ, Δχ, and Ω, respectively; and $\sigma_{\Delta S_{mix}}^{2}$, $\sigma_{\Delta H_{mix}}^{2}$, $\sigma_{\delta }^{2}$, $\sigma_{\Delta \chi }^{2}$, and $\sigma_{\Omega }^{2}$ are the variance of $\Delta S_{mix}$, $\Delta H_{mix}$, δ, Δχ, and Ω, respectively, in each dataset. The candidate algorithm set, $C=\{C_{1},C_{2},\ldots ,C_{M}\}$, includes the decision tree (DT), KNN, SVM, random forest (RF), and bagging. The meta-knowledge table describes the relationship between the values of meta-features (*MF*) and the accuracy of candidate algorithms (*CA*).

The new meta-learner based on SNN and DISKR is proposed to improve the performance of meta-learning and is called improved SNN and DISKR (ISD). Therefore, an MLRM based on improved meta-learning is proposed. The meta-model is the trained meta-learner, ISD.

The steps of the MLRM are as follows:

Input: Data library $L=\{D_{1},D_{2},\ldots ,D_{N}\}$, Candidate algorithm set $C=\{C_{1},C_{2},\ldots ,C_{M}\}$, New dataset *D_new_*.

Output: Recommendation algorithm.

Step 1: Compute the parameters $\Delta S_{mix}$, $\Delta H_{mix}$, δ, Δχ, and Ω of each sample in each dataset, *D_i_*, by Equations (1)–(5) in Section 2.1.

Step 2: Construct meta-features and calculate the values of meta-features: $\bar{\Delta S_{mix}}$, $\bar{\Delta H_{mix}}$, $\bar{\delta }$, $\bar{\Delta \chi }$, $\bar{\Omega }$, $\sigma_{\Delta S_{mix}}^{2}$, $\sigma_{\Delta H_{mix}}^{2}$, $\sigma_{\delta }^{2}$, $\sigma_{\Delta \chi }^{2}$, and $\sigma_{\Omega }^{2}$.

Step 3: Based on the values of meta-features and the real classification results on each dataset, train candidate algorithms DT, KNN, SVM, RF, and bagging in candidate algorithm set $C=\{C_{1},C_{2},\ldots ,C_{M}\}$ to evaluate their accuracies.

Step 4: The meta-knowledge table is established based on meta-features of datasets and accuracies of candidate algorithms. The values of meta-features are used as the input variable (*MF*). The accuracies of candidate algorithms are used as the output variable (*CA*).

Step 5: Train the new meta-learner, ISD, based on the input variable, *MF*, and output variable, *CA*, in Step 4. Thus, obtain the trained meta-learner ISD, also known as the meta-model.

Step 6: Compute parameters of each sample in the new dataset, *D_new_*, by the parameter method: $\Delta S_{mix}$, $\Delta H_{mix}$, δ, Δχ, and Ω. Compute the values of meta-features, *MF_new_*, of the new dataset, *D_new_*: $\bar{\Delta S_{mix}}$, $\bar{\Delta H_{mix}}$, $\bar{\delta }$, $\bar{\Delta \chi }$, $\bar{\Omega }$, $\sigma_{\Delta S_{mix}}^{2}$, $\sigma_{\Delta H_{mix}}^{2}$, $\sigma_{\delta }^{2}$, $\sigma_{\Delta \chi }^{2}$, and $\sigma_{\Omega }^{2}$.

Step 7: Input meta-features, *MF_new_*, of the new dataset, *D_new_*, to the meta-model. The realization process of the meta-model is as follows.

(1) The nearest neighbor set, $SNN(MF_{new})$, for the meta-features, *MF_new_*, of the new dataset, *D_new_*, is obtained by SNN in Section 2.3 based on the meta-knowledge table.
(2) The subset, *S*, is obtained by DISKR in Section 2.4, which is the remaining sample on the meta-knowledge table after removing outliers and points that have less effect on KNN. The nearest neighbor set, $DI(MF_{new})$, for the meta-features, *MF_new_*, is obtained by the first *k* samples with the smallest distance in DISKR.
(3) Obtain the nearest neighbor set, $SD(MF_{new})$, for the meta-features, *MF_new_*, of the new dataset, *D_new_*, by ISD. The nearest neighbor set, $SD(MF_{new})$, is shown as Equation (15):
  (15) $$SD(MF_{new})=SNN(MF_{new})\cap DI(MF_{new})$$
  where the nearest neighbor set, $SD(MF_{new})$, is obtained by the intersection of $SNN(MF_{new})$ and $DI(MF_{new})$.

Step 8: Output the average accuracies of candidate algorithms by $SD(MF_{new})$. Select the algorithm with the highest average accuracy as the recommended algorithm.

### 3.2. Phase Prediction of HEAs

The criterion of the SS phase has been verified by a large number of experiments in the material field. In the criterion of SS, if δ and Ω fall within the range of δ≤6.6% and Ω≥1.1, the SS phase is easily formed [5,11,13]. The decision process of the criterion is easy to understand, saves computation time, and has strong interpretability and generalization. In this paper, the criterion of SS is integrated into the phase prediction of HEAs.

Combining the advantages of strong learning ability of MLRM and strong interpretability and generalization of the criterion of SS, the phase prediction of HEAs is proposed by integrating criterion and MLRM. The PPH is a serial model. First, the criterion of the SS phase is used to determine the phases of the test dataset, *T*. Secondly, when the remaining samples cannot be determined by the criterion of SS, the MLRM is adopted to recommend the appropriate ML algorithm for the remaining samples. Finally, the recommended algorithm is used to predict the phase of HEAs.

**Figure 4:**
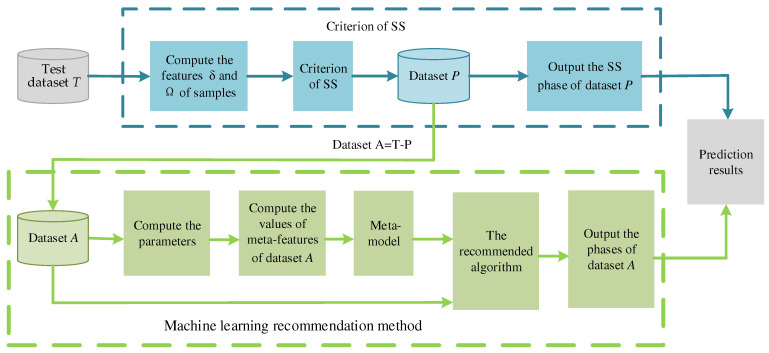
Schematic diagram of phase prediction frame of HEAs.

A schematic diagram of the PPH is shown in Figure 4.

In Figure 4, the blue frame is the criterion of SS, and the green frame is the MLRM. The specific details of the PPH are illustrated as follows:

Input: Test dataset $T=\{t_{1},t_{2},\ldots t_{N}\}$.

Output: Phases of samples in *T*.

Step 1: Compute the features δ and Ω of samples in test dataset *T*. Utilize the criterion of SS to judge the phase of samples. The dataset, $P=\{p_{1},p_{2},\ldots ,p_{J}\}$, is composed of samples satisfying δ≤6.6% and Ω≥1.1. Output: the judgement results of samples in the dataset, *P*, are SS.

Step 2: In addition to the samples of dataset *P*, the remaining samples of the test dataset, *T*, constitute dataset $A=\{a_{1},a_{2},\ldots ,a_{Q}\}$, that is, *A* = *T* − *P*. When the criterion of SS cannot judge the samples of dataset *A*, the machine learning recommendation method is adopted to predict phases of HEAs.

Step 3: For dataset *A*, compute the parameters of each sample in dataset *A* by Equations (1)–(5) in Section 2.1, and compute the values of meta-features of dataset *A*. Then, input the values of meta-features into the meta-model.

Step4: Predict the accuracies of the candidate algorithms by meta-model, and select an ideal algorithm as the recommended algorithm in dataset *A*.

Step5: The recommended algorithm is used to predict the phase of dataset *A*.

Step6: Output the phases of test dataset *T*, combining results of dataset *P* and dataset *A*.

## 4. Results and Discussions

In order to verify the effectiveness of the proposed PPH, numerous experiments are carried out. The accuracy of the proposed method is compared with several traditional ML algorithms, including DT, KNN, SVM, RF, and bagging [41,42,43]. In this paper, the parameters of the algorithms in this experiment are introduced. In DT, the hyperparameter ‘maximum depth’ is set to ‘25’. The hyperparameter ‘*k*’ of KNN is set to ‘5’. The support vector classification (SVC) method is used in SVM. In bagging, the hyperparameter ‘number of weak learners’ is set to ‘11’. In ISDISKR, the hyperparameter ‘θ’ is set as ‘0.05’. The other parameters of all ML algorithms above are set as default.

The experiments are carried out by the sklearn library of Python (version 3.7.4). Python was created by Guido van Rossum in Amsterdam, the Netherlands. The hardware device is a laptop, and the brand of the computer is Lenovo. The CPU is an Intel(R) Core(M) I7-8700 CPU @ 3.20 GHz 3.19 GHz, the RAM is 16.0 GB, the manufacturer is Lenovo Group Limited, and the location is Beijing, China.

### 4.1. Overview of Experimental Datasets

**Figure 5:**
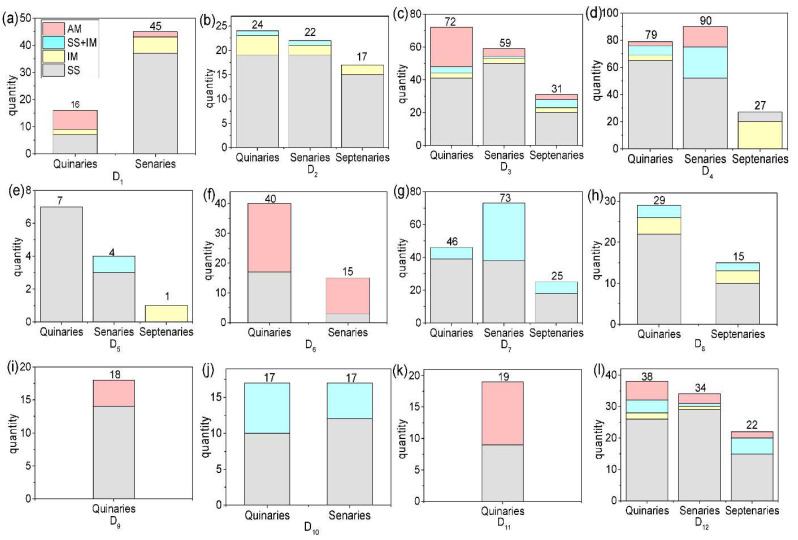
Quantity of SS, IM, SS+IM, and AM in the quinaries, senaries, and septenaries. (**a**) D1, (**b**) D2, (**c**) D3, (**d**) D4, (**e**) D5, (**f**) D6, (**g**) D7, (**h**) D8, (**i**) D9, (**j**) D10, (**k**) D11, and (**l**) D12.

In our study, the 12 HEAs datasets were collected from several papers [4,6,7,10,14,44,45,46,47,48,49]. For convenience, the 12 datasets are sequentially marked by D_1_, D_2_, D_3_, D_4_, D_5_, D_6_, D_7_, D_8_, D_9_, D_10_, D_11_, and D_12_. The 12 datasets contain 902 samples, including 405 quinary HEAs, 359 senary HEAs, and 138 septenary HEAs. The phases of 902 samples include SS, IM, SS+IM, and AM. The quantity of SS, IM, SS+IM, and AM of the quinaries, senaries, and septenaries in 12 datasets is shown in Figure 5. As shown in Figure 5, the number of SS is the largest, and the number of IM is the least among the 12 datasets. Figure 5 shows that each dataset contains different quantities of quinaries, senaries, and septenaries.

**Figure 6:**
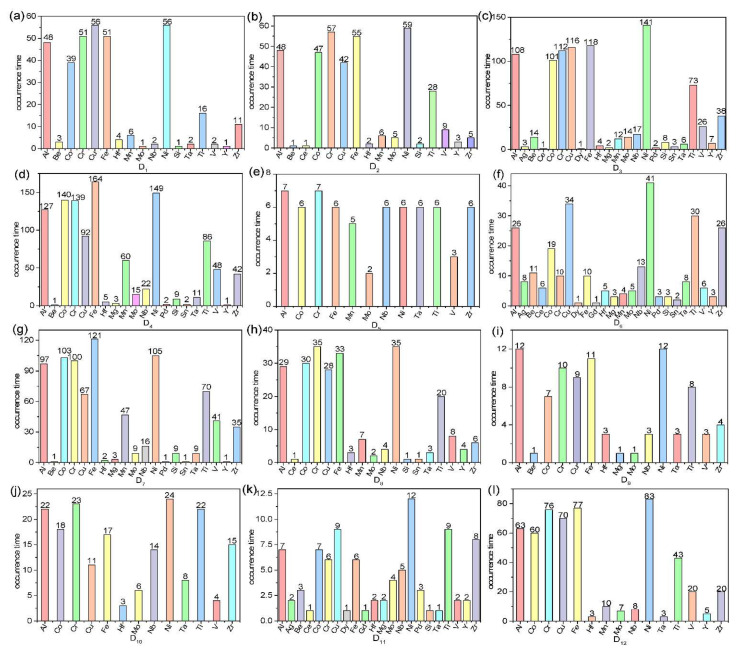
Statistics of the occurrence time of each element in 12 datasets. (**a**) D1, (**b**) D2, (**c**) D3, (**d**) D4, (**e**) D5, (**f**) D6, (**g**) D7, (**h**) D8, (**i**) D9, (**j**) D10, (**k**) D11, (**l**) D12.

The 12 datasets include 24 elements, which contain Al, Ag, Be, Ce, Co, Cr, Cu, Dy, Fe, Gd, Hf, Mg, Mn, Mo, Nb, Ni, Pd, Si, Sn, Ta, Ti, V, Y, and Zr. The occurrence time of each element in each dataset is shown in Figure 6. As shown in Figure 6, the occurrence time of different elements varies. The elements Al, Co, Cr, Cu, Fe, Ni, and Ti appear more frequently, and the element Gd appears only once in 12 datasets.

The maximum and minimum values of the five input parameters, $\Delta S_{mix}$, $\Delta H_{mix}$, δ, Δχ, and Ω, are shown in Table 1.

A snapshot of part of the samples in the HEA dataset according to the Pandas data frame format is shown as Table 2. In Table 2, the first column is the number of HEAs, and the second column provides the names of the HEAs. The columns ranging from three to seven are the parameters of HEAs, i.e., δ, $\Delta H_{mix}$, Ω, Δχ, and $\Delta S_{mix}$. The final column is the phases of HEAs. Table 2 shows a snapshot of the first six samples taken from all HEAs.

**Figure 7:**
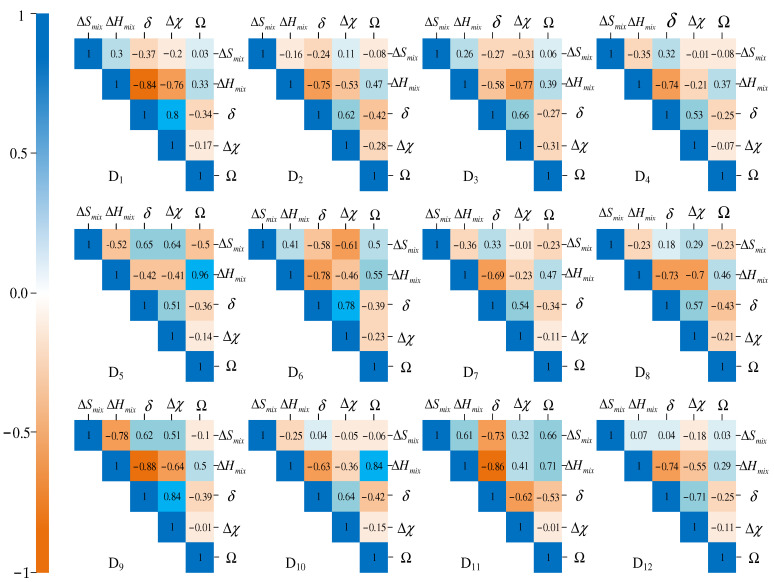
Heatmap of Pearson correlation coefficient matrix among 5 parameters in 12 datasets.

The correlation between any two parameters in Table 2 plays an important role in phase prediction of HEAs, so the Pearson correlation coefficient, *C_ab_*, is selected to describe the correlations of HEA parameters, as shown in Equation (16) [14]:

(16) $$C_{ab}=\frac{1}{n-1}\frac{\sum_{i=1}^{n}\left(a_{i}-\bar{a})(b_{i}-\bar{b}\right)}{s_{a}s_{b}}$$

where *a_i_* and *b_i_* are the sample values of parameters *a* and *b*, respectively; $\bar{a}$ and $\bar{b}$ are the mean values of parameters *a* and *b*, respectively; *s_a_* and *s_b_* are the standard deviation of parameters *a* and *b*, respectively; and *n* is the number of samples. If $C_{ab}=1$ or $C_{ab}=-1$, the parameters *a* and *b* are almost completely relevant or completely irrelevant, respectively. The correlation among the 5 parameters of 12 datasets is shown in Figure 7.

In Figure 7, the values of the correlation coefficient between the two corresponding parameters is shown in every grid. Color intensity is proportional to the correlation coefficients. In the left side of the correlogram, the legend color shows the relationship between the correlation coefficients and the corresponding color. The darker the blue color, the higher the correlation; the darker the orange color, the lower the correlation. The range of *C_ab_* in 12 datasets is between−0.88 and 0.96. Among them, ∆*H_mix_* and Ω in D_5_ have the highest correlation.

### 4.2. Comparison between Meta-Learning and Traditional ML Algorithms

**Figure 8:**
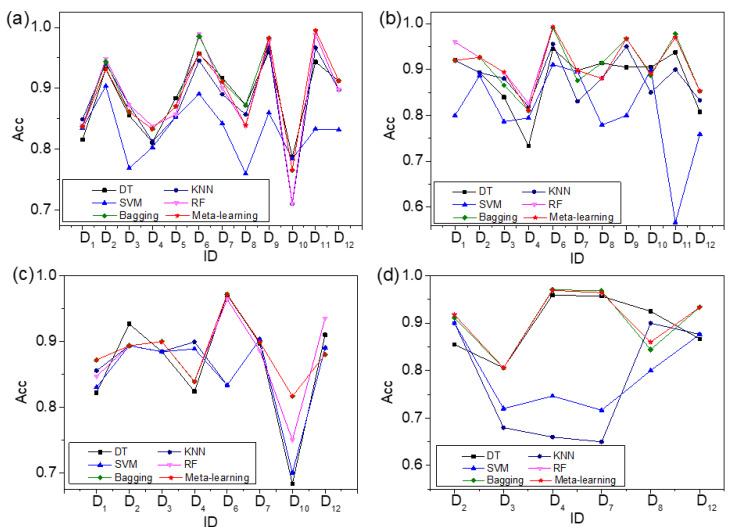
Accuracy comparison between meta-learning and traditional ML algorithms. (**a**) all, (**b**) quinaries, (**c**) senaries, and (**d**) septenaries.

The meta-learning based on meta-knowledge table can recommend an ideal algorithm for material designers so as to predict the phases of HEAs. In order to validate the performance of meta-learning, the experimental results of meta-learning are compared with the DT, KNN, SVM, RF, and bagging [41,42,43]. The fivefold cross-validation method is also carried out 20 times to avoid the overfitting problem of ML algorithms [50]. The comparison experimental results between meta-learning and traditional ML algorithms for all, quinaries, senaries, and septenaries are shown in Figure 8. In Figure 8, the experimental results of all, quinaries, senaries, and septenaries between meta-learning and other traditional ML algorithms are shown in (a), (b), (c), and (d), respectively. In Figure 8a, “all” represents samples containing quinaries, senaries, and septenaries. The horizontal axis represents the ID of these datasets, and the vertical axis represents the accuracies of meta-learning and traditional ML algorithms. The red line with pentagonal stars represents the accuracy of algorithms recommended by meta-learning. The black line with squares shows the accuracy of DT. The navy line with circles shows the accuracy of KNN. The blue line with upward triangles represents the accuracy of SVM. The pink line with downward triangles shows the accuracy of RF, and the olive line with diamonds represents the accuracy of bagging. Based on the results presented in Figure 8, the algorithm with the highest accuracy can be recommended by meta-learning on some datasets.

In order to facilitate the reader’s observation, the results of all, quinaries, senaries, and septenaries are shown in Table 3, Table 4, Table 5 and Table 6. The bold part of the tables is the algorithm with the highest accuracy on the corresponding dataset.

In Table 3, the first column is the ID of 12 datasets, and the second column to the seventh column are the accuracies of DT, KNN, SVM, RF, bagging, and meta-learning, respectively. As shown in Table 3, meta-learning recommends the algorithm with the highest accuracy; in D_9_, D_11_, and D_12_, the algorithms with the highest accuracy are bagging, bagging, and bagging, and the accuracies are 0.983, 0.995, and 0.912, respectively, for all. In D_4_, D_5_, and D_7_, although meta-learning does not recommend the highest-accuracy algorithm, it recommends the algorithm with the second highest accuracy; the algorithms with the second highest accuracy are bagging, bagging, and bagging, and the accuracies are 0.833, 0.871, 0.911, respectively, for all.

It can be seen from Table 4 that meta-learning recommends the algorithm with the highest accuracy in D_2_, D_3_, D_6_, D_7_, D_9_, and D_12_, and the accuracies are 0.927, 0.894, 0.994, 0.898, 0.968, 0.853, respectively, for quinaries. Meta-learning recommends the algorithm with the second highest accuracy in D_1_ and D_11_; the algorithms with the second highest accuracy are bagging and RF in D_1_ and D_11_, respectively.

As shown in Table 5, meta-learning recommends the algorithm with the highest accuracy in D_1_, D_3_, D_6_, and D_10_ for senaries, and the accuracies are 0.872, 0.900, 0.972, 0.817, respectively. Meta-learning recommends the algorithm with the second highest accuracy in D_2_ for senaries; the algorithm with the second highest accuracy is bagging in D_2_.

In Table 6, the algorithm with the highest accuracy is recommended by meta-learning in D_2_, D_3_, and D_12_ for septenaries; the algorithms with the highest accuracy are RF, RF, and RF in D_2_, D_3_, and D_12_, respectively. Meta-learning recommends the algorithm with the second highest accuracy in D_4_ and D_7_ for septenaries; the accuracies are 0.969 and 0.964, respectively.

Meta-learning recommends the algorithm with the high highest accuracy in some datasets. The reason should be that meta-learning can mine the relationship between mathematical statistical characteristics of HEA datasets and the performance of algorithms. The mathematical statistical characteristics of datasets include the mean value and variance of these parameters presented in Section 2.1. It is similarly reported in the literature in other fields meta-learning can recommend the desirable algorithm in most cases [24,27]. The experimental results also show that RF and bagging have higher accuracies than DT, KNN, and SVM [41,42,43]. The reason could be that RF and bagging are both ensemble algorithms, which are composed of multiple classifiers and integrate all the classification results.

However, meta-learning cannot recommend the algorithm with the highest accuracy in some datasets. The reasons could be as follows: firstly, the meta-learner is the crucial part in meta-learning method, and the meta-learner of meta-learning is KNN. The performance of KNN is not ideal in ML algorithms. Secondly, the KNN does not fully consider the information of the left and right nearest neighbors of every sample by every attribute. Finally, the meta-learner, KNN, does not consider how to exclude the noise samples and make up for missing information, which influences the decision result of different algorithms. If the performance of the meta-learner can be improved, the recommendation of meta-learning will be more accurate.

### 4.3. Comparison between MLRM and Meta-Learning

**Figure 9:**
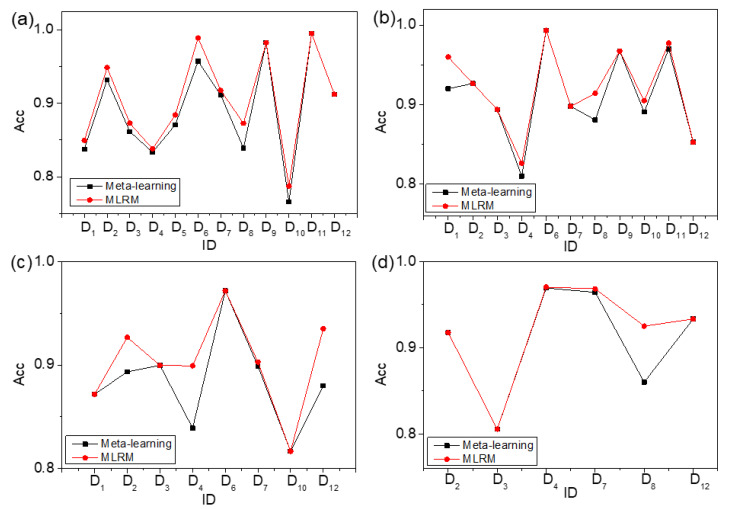
Accuracy comparison between MLRM and meta-learning. (**a**) all, (**b**) quinaries, (**c**) senaries, and (**d**) septenaries.

To alleviate the problem presented in Section 4.2, a novel MLRM method is proposed based on improved meta-learning. In order to validate the performance of MLRM, a comparison between MLRM and meta-learning is shown in Figure 9. In Figure 9, the accuracies of MLRM and meta-learning of all, quinaries, senaries, and septenaries are shown in (a), (b), (c), and (d), respectively. The horizontal axis is the ID of these datasets, and the vertical axis is the accuracy. The red line with circles shows the accuracy of algorithms recommended by MLRM. The black line with squares represents the accuracy of algorithms recommended by meta-learning. It can be seen from Figure 9 that all experimental results of MLRM are better than those of meta-learning.

In order to facilitate the reader’s observation, the rankings of algorithms recommended by meta-learning and MLRM, as wells real rankings are listed in Table 7, Table 8, Table 9 and Table 10. The accuracies of the optimal algorithms recommended by meta-learning and MLRM, as well as the accuracies of real optimal algorithms are listed in Table 11, Table 12, Table 13 and Table 14.

In Table 7, the first column is the ID of datasets. The second column and the third column are the ranking of the algorithms recommended by meta-learning and MLRM, respectively. The fourth column is the real algorithm ranking, which is also called TURE. The previous two best algorithms recommended by meta-learning and MLRM are listed from column 2 to column 3. The fourth column represents the actual previous two best algorithms. The first algorithm name and the second algorithm name appearing from column 2 to column 4 represent the highest and the second highest algorithm. In Table 11, the first column is the ID of datasets, and the second column to the third column are the accuracies of the optimal algorithms recommended by meta-learning and MLRM. The fourth column is the accuracy of the real optimal algorithm. As seen in Table 7 and Table 11, the MLRM can recommend the algorithm with the highest accuracy in 12 datasets for all. However, meta-learning only recommends the algorithm with the highest accuracy in D_9_, D_11_, and D_12_; the second highest accuracy in D_4_, D_5_, and D_7_; and neither the highest nor the second highest accuracy algorithm in other datasets.

According to Table 8 and Table 12, meta-learning only recommends the algorithm with the highest accuracy in D_2_, D_3_, D_6_, D_7_, D_9_, and D_12_; and the second highest accuracy in D_1_ and D_11_. The MLRM can recommend the algorithm with the highest accuracy in every dataset for quinaries.

As shown in Table 9 and Table 13, MLRM recommends the algorithm with the highest accuracy for senaries. However, meta-learning only recommends the algorithm with the highest accuracy in D_1_, D_3_, D_6_, and D_10_; and the second highest accuracy in D_2_. The quantity of ideal algorithms recommended by meta-learning is less than that recommended by MLRM.

As shown in Table 10 and Table 14, meta-learning only recommends the algorithm with the highest accuracy in D_2_, D_3_, and D_12_; and the second highest accuracy in D_4_ and D_7_. The MLRM recommends the algorithm with the highest accuracy in 12 datasets for septenaries.

In summary, MLRM can recommend the algorithm with the highest accuracy in every dataset, whereas meta-learning can only recommend the algorithm with the highest accuracy on several datasets. The reasons may be that the meta-learner in traditional meta-learning is KNN, and the meta-learner in MLRM is ISD based on DISKR and SNN. KNN performs poorly when there are noise points and does not consider the useful neighbor information of each sample. In other fields, the DISKR in MLRM has been shown to remove noise points and reduce the size of the datasets to improve performance [30]. The SNN in MLRM is adopted to obtain more reliable and useful left and right neighbor information [31].

### 4.4. Comparison between PPH and MLRM

**Figure 10:**
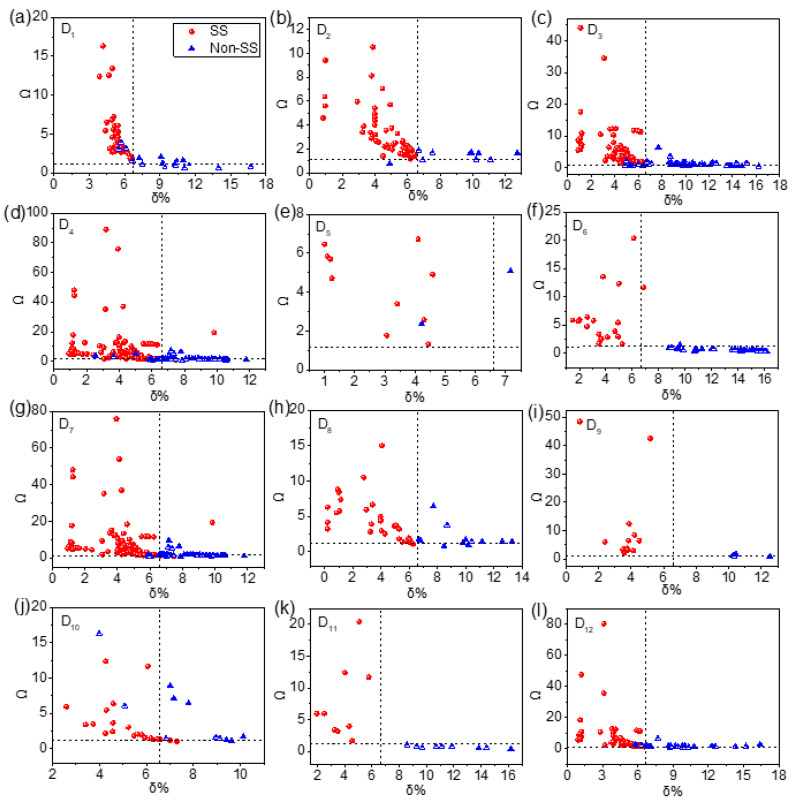
Scatter plot based on the δ−Ω coordinate system. (**a**) D1, (**b**) D2, (**c**) D3, (**d**) D4, (**e**) D5, (**f**) D6, (**g**) D7, (**h**) D8, (**i**) D9, (**j**) D10, (**k**) D11, (**l**) D12.

A PPH is proposed based on the criterion of SS and MLRM. The criterion of SS is as follows: if the δ and Ω of samples are in the scope of δ≤6.6% and Ω≥1.1, the phases of samples are recognized as SS. To better illustrate the criterion of SS, a scatter plot based on the δ−Ω coordinate system is shown in Figure 10, which shows the distribution of SS and non-SS of HEAs. In Figure 10, the horizontal axis is δ%, and the vertical axis is Ω. The horizontal virtual line is Ω=1.1, the vertical virtual line is δ=6.6%, the upper left corner of the dotted line represents the scope of δ≤6.6%, and Ω≥1.1. The red circle represents the samples for which the phase is SS, and the blue triangle represents the samples for which the phase is non-SS. Figure 10 shows that the phase of most samples in the upper left corner are SS in each dataset, and most samples of SS fall within the range of δ≤6.6% and Ω≥1.1. The experimental results show that the criterion δ≤6.6% and Ω≥1.1 has good effect on SS phase determination. Therefore, the criterion of SS is integrated to the PPH to improve the phase prediction accuracy of HEAs.

**Figure 11:**
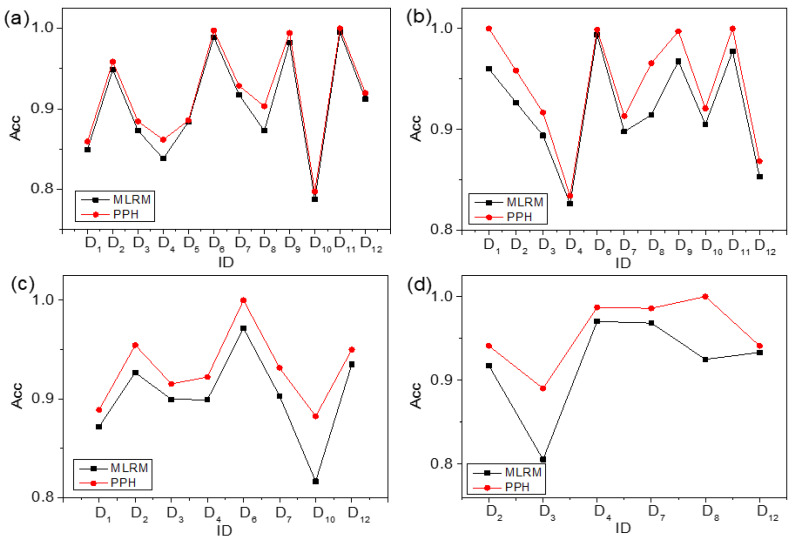
Accuracy comparison between PPH and MLRM. (**a**) all, (**b**) quinaries, (**c**) senaries, and (**d**) septenaries.

In order to verify the performance of the PPH, a set of comparison experiments between PPH and MLRM are carried out. The comparison of the results is shown in Figure 11. In Figure 11, the accuracies of PPH and MLRM in all, quinaries, senaries, and septenaries are shown in (a), (b), (c), and (d), respectively. The horizontal axis is the ID of these datasets, and the vertical axis is the accuracy. The red line with circles shows the accuracy of PPH. The black line with squares represents the accuracy of algorithms recommended by MLRM. As shown in Figure 11, PPH has higher prediction accuracy than MLRM in all, quinaries, senaries, and septenaries.

The specific results are listed in Table 15. The first column is the ID of datasets. Column 2, column 4, column 6, and column 8 are the accuracies of MLRM in all, quinaries, senaries, and septenaries, respectively. Column 3, column 5, column 7, and column 9 are the accuracies of PPH in all, quinaries, senaries, and septenaries, respectively. NULL represents no accuracy of MLRM and PPH. From Table 15, it can be concluded that the accuracy of PPH is higher than that of MLRM. The reason could be that PPH combines the advantages of the criterion of SS and MLRM. The MLRM is a pure ML method, and it does not make full use of the professional knowledge in the material field. The criterion of SS has strong interpretability and generalization and requires only a small amount of training samples. Therefore, the prediction accuracy of PPH is higher than that of MLRM [33,34,51].

## 5. Conclusions

In order to predict the phases of HEAs, in this paper, we propose the phase prediction of HEAs by integrating the criterion and machine learning recommendation method. First, a meta-knowledge table based on characteristics of HEAs and performance of candidate algorithms is established, and the experiments show that meta-learning can recommend the algorithm with ideal accuracy on some datasets for material designers. Second, in order to guide material designers to select an algorithm with higher accuracy, an MLRM based on SNN and DISKR is proposed. The experimental results show that the recommendation accuracy of the MLRM is higher than that of meta-learning based on KNN on all, quinary, senary, and septenary HEAs. Third, the PPH consisting of the criterion of SS and MLRM is proposed. Compared with other ML algorithms in the HEA field, the experimental results show that the PPH can achieve performance than the traditional meta-learning method. The average prediction accuracy of the PPH in all, quinary, senary, and septenary HEAs is 91.6%, 94.3%, 93.1%, and 95.8%, respectively. The method proposed in this paper can reduce the burden of material designers in selecting algorithms, effectively improve the prediction accuracy of HEAs phase, and considerably reduce the experimental time and cost. In addition, the PPH can also provide a research foundation in other material fields.

## Figures and Tables

**Table 1:** Maximum values and minimum values of the five parameters.

Number	Parameter	Maximum Value	Minimum Value	Parameter Description
1	$\Delta S_{mix}$	16.18	7.78	thermodynamic parameter
2	$\Delta H_{mix}$	17.12	−48.64	chemical parameter
3	δ	35.30	0.21	electronic parameter
4	Δχ	23.10	0.04	electronic parameter
5	Ω	283.50	0.37	chemical thermodynamic parameter

**Table 2:** Pandas snapshot of the first six samples.

Number	HEAs	$\Delta S_{mix}$	$\Delta H_{mix}$	δ(%)	Δχ	Ω	Phase
0	AlCr0.5NbTiV	13.150000	−15.410000	5.230000	0.037647	1.680000	SS
1	Mg65Cu15Ag5Pd5Gd10	9.100000	−13.240000	9.360000	0.298062	0.770000	AM
2	AlCoCrFeNiSi0.6	14.778118	−22.755102	5.877203	0.120010	1.090691	SS+IM
3	CoFeMnTiVZr0.4	14.585020	−16.049383	8.088626	0.165697	1.692345	IM
4	Ti0.2CoCrFeNiCuAl0.5	15.445251	−4.148969	0.210000	0.118750	6.318158	SS
5	Al0.5CoCrCuFeNiTi0.8	15.995280	−10.100000	5.800000	0.137280	2.725683	SS+IM

**Table 3:** Accuracy comparison between meta-learning and traditional ML algorithms in all.

ID	DT	KNN	SVM	RF	Bagging	Meta-Learning
D_1_	0.816 ± 0.012	**0.849 ± 0.008**	0.835 ± 0.007	0.839 ± 0.010	0.837 ± 0.005	0.837 ± 0.005
D_2_	0.932 ± 0.003	0.941 ± 0.022	0.904 ± 0.009	**0.949 ± 0.013**	0.944 ± 0.020	0.932 ± 0.003
D_3_	0.856 ± 0.008	0.873 ± 0.019	0.769 ± 0.013	**0.873 ± 0.003**	0.861 ± 0.002	0.861 ± 0.002
D_4_	0.811 ± 0.005	0.813 ± 0.012	0.803 ± 0.017	**0.838 ± 0.009**	0.833 ± 0.014	0.833 ± 0.014
D_5_	**0.884 ± 0.030**	0.853 ± 0.016	0.853 ± 0.011	0.857 ± 0.012	0.871 ± 0.030	0.871 ± 0.030
D_6_	0.957 ± 0.009	0.945 ± 0.004	0.891 ± 0.007	**0.989 ± 0.007**	0.985 ± 0.008	0.957 ± 0.009
D_7_	**0.918 ± 0.010**	0.890 ± 0.027	0.842 ± 0.005	0.900 ± 0.008	0.911 ± 0.009	0.911 ± 0.009
D_8_	**0.873 ± 0.025**	0.857 ± 0.017	0.760 ± 0.023	0.839 ± 0.011	0.872 ± 0.006	0.839 ± 0.011
D_9_	0.960 ± 0.003	0.967 ± 0.006	0.860 ± 0.009	0.978 ± 0.010	**0.983 ± 0.010**	**0.983 ± 0.010**
D_10_	**0.787 ± 0.009**	0.711 ± 0.019	0.784 ± 0.010	0.712 ± 0.012	0.766 ± 0.026	0.766 ± 0.026
D_11_	0.943 ± 0.006	0.967 ± 0.016	0.833 ± 0.022	0.988 ± 0.002	**0.995 ± 0.002**	**0.995 ± 0.002**
D_12_	0.912 ± 0.004	0.898 ± 0.012	0.832 ± 0.009	0.898 ± 0.015	**0.912 ± 0.003**	**0.912 ± 0.003**

**Table 4:** Accuracy comparison between meta-learning and traditional ML algorithms in quinaries.

ID	DT	KNN	SVM	RF	Bagging	Meta-Learning
D_1_	0.920 ± 0.005	0.920 ± 0.003	0.800 ± 0.002	**0.960 ± 0.027**	0.920 ± 0.002	0.920 ± 0.002
D_2_	0.893 ± 0.015	0.893 ± 0.012	0.887 ± 0.009	0.927 ± 0.005	**0.927 ± 0.004**	**0.927 ± 0.004**
D_3_	0.840 ± 0.005	0.881 ± 0.015	0.786 ± 0.017	**0.894 ± 0.010**	0.866 ± 0.010	**0.894 ± 0.010**
D_4_	0.733 ± 0.034	0.819 ± 0.011	0.795 ± 0.035	**0.826 ± 0.034**	0.810 ± 0.008	0.810 ± 0.008
D_6_	0.945 ± 0.025	0.956 ± 0.005	0.911 ± 0.005	**0.994 ± 0.002**	0.991 ± 0.001	**0.994 ± 0.002**
D_7_	0.898 ± 0.007	0.831 ± 0.004	0.895 ± 0.003	**0.898 ± 0.005**	0.876 ± 0.002	**0.898 ± 0.005**
D_8_	**0.914 ± 0.004**	0.881 ± 0.007	0.779 ± 0.016	0.881 ± 0.003	0.914 ± 0.006	0.881 ± 0.003
D_9_	0.905 ± 0.027	0.950 ± 0.011	0.800 ± 0.022	**0.968 ± 0.006**	0.968 ± 0.012	**0.968 ± 0.006**
D_10_	**0.905 ± 0.004**	0.850 ± 0.008	0.900 ± 0.003	0.891 ± 0.027	0.888 ± 0.007	0.891 ± 0.027
D_11_	0.938 ± 0.006	0.900 ± 0.024	0.567 ± 0.027	0.970 ± 0.009	**0.978 ± 0.005**	0.970 ± 0.009
D_12_	0.808 ± 0.025	0.833 ± 0.023	0.759 ± 0.029	**0.853 ± 0.022**	0.853 ± 0.033	**0.853 ± 0.022**

**Table 5:** Accuracy comparison between meta-learning and traditional ML algorithms in senaries.

ID	DT	KNN	SVM	RF	Bagging	Meta-Learning
D_1_	0.822 ± 0.015	0.855 ± 0.008	0.830 ± 0.014	0.847 ± 0.007	**0.872 ± 0.006**	**0.872 ± 0.006**
D_2_	**0.927 ± 0.009**	0.893 ± 0.013	0.893 ± 0.009	0.893 ± 0.020	0.893 ± 0.006	0.893 ± 0.006
D_3_	0.884 ± 0.008	0.884 ± 0.011	0.884 ± 0.018	0.900 ± 0.014	**0.900 ± 0.013**	**0.900 ± 0.013**
D_4_	0.824 ± 0.012	**0.899 ± 0.017**	0.889 ± 0.005	0.839 ± 0.010	0.838 ± 0.017	0.839 ± 0.010
D_6_	0.971 ± 0.010	0.833 ± 0.009	0.833 ± 0.011	0.964 ± 0.017	**0.972 ± 0.012**	**0.972 ± 0.012**
D_7_	0.897 ± 0.007	**0.903 ± 0.002**	0.903 ± 0.013	0.886 ± 0.008	0.899 ± 0.008	0.899 ± 0.008
D_10_	0.683 ± 0.023	0.700 ± 0.036	0.700 ± 0.010	0.750 ± 0.032	**0.817 ± 0.006**	**0.817 ± 0.006**
D_12_	0.910 ± 0.020	0.890 ± 0.007	0.890 ± 0.009	**0.935 ± 0.005**	0.880 ± 0.027	0.880 ± 0.027

**Table 6:** Accuracy comparison between meta-learning and traditional ML algorithms in septenaries.

ID	DT	KNN	SVM	RF	Bagging	Meta-Learning
D_2_	0.855 ± 0.031	0.900 ± 0.005	0.900 ± 0.007	**0.918 ± 0.022**	0.911 ± 0.013	**0.918 ± 0.022**
D_3_	0.806 ± 0.016	0.680 ± 0.008	0.720 ± 0.005	**0.806 ± 0.007**	0.806 ± 0.011	**0.806 ± 0.007**
D_4_	0.959 ± 0.009	0.660 ± 0.028	0.747 ± 0.012	0.969 ± 0.013	**0.970 ± 0.005**	0.969 ± 0.013
D_7_	0.957 ± 0.015	0.650 ± 0.019	0.717 ± 0.019	0.964 ± 0.009	**0.968 ± 0.018**	0.964 ± 0.009
D_8_	**0.925 ± 0.033**	0.900 ± 0.023	0.800 ± 0.035	0.860 ± 0.018	0.844 ± 0.017	0.860 ± 0.018
D_12_	0.867 ± 0.027	0.876 ± 0.034	0.876 ± 0.031	**0.933 ± 0.022**	0.933 ± 0.033	**0.933 ± 0.022**

**Table 7:** Recommendation results between MLRM and meta-learning in all.

ID	Meta-Learning	MLRM	TRUE
D_1_	Bagging, KNN	KNN, RF	KNN, RF
D_2_	DT, Bagging	RF, Bagging	RF, Bagging
D_3_	Bagging, RF	RF, Bagging	RF, KNN
D_4_	Bagging, DT	RF, Bagging	RF, Bagging
D_5_	Bagging, RF	DT, Bagging	DT, Bagging
D_6_	DT, Bagging	RF, Bagging	RF, Bagging
D_7_	Bagging, RF	DT, Bagging	DT, Bagging
D_8_	RF, Bagging	DT, Bagging	DT, Bagging
D_9_	Bagging, DT	Bagging, RF	Bagging, RF
D_10_	Bagging, RF	DT, Bagging	DT, SVM
D_11_	Bagging, RF	Bagging, RF	Bagging, RF
D_12_	Bagging, RF	Bagging, RF	Bagging, DT

**Table 8:** Recommendation results between MLRM and meta-learning in quinaries.

ID	Meta-Learning	MLRM	TRUE
D_1_	Bagging, RF	RF, Bagging	RF, Bagging
D_2_	Bagging, RF	Bagging, RF	Bagging, RF
D_3_	RF, Bagging	RF, Bagging	RF, KNN
D_4_	Bagging, RF	RF, Bagging	RF, KNN
D_6_	RF, Bagging	RF, Bagging	RF, Bagging
D_7_	RF, Bagging	RF, Bagging	RF, DT
D_8_	RF, Bagging	DT, Bagging	DT, Bagging
D_9_	RF, Bagging	RF, Bagging	RF, Bagging
D_10_	RF, Bagging	DT, RF	DT, SVM
D_11_	RF, Bagging	Bagging, RF	Bagging, RF
D_12_	RF, Bagging	RF, Bagging	RF, Bagging

**Table 9:** Recommendation results between MLRM and meta-learning in senaries.

ID	Meta-Learning	MLRM	TRUE
D_1_	Bagging, RF	Bagging, KNN	Bagging, KNN
D_2_	Bagging, RF	DT, Bagging	DT, Bagging
D_3_	Bagging, RF	Bagging, RF	Bagging, RF
D_4_	RF, Bagging	KNN, SVM	KNN, SVM
D_6_	Bagging, RF	Bagging, RF	Bagging, DT
D_7_	Bagging RF	KNN, SVM	KNN, SVM
D_10_	Bagging, DT	Bagging, RF	Bagging, RF
D_12_	Bagging RF	RF, DT	RF, DT

**Table 10:** Recommendation results between MLRM and meta-learning in septenaries.

ID	Meta-Learning	MLRM	TRUE
D_2_	RF, Bagging	RF, Bagging	RF, Bagging
D_3_	RF, Bagging	RF, Bagging	RF, Bagging
D_4_	RF, Bagging	Bagging, RF	Bagging, RF
D_7_	RF, Bagging	Bagging, RF	Bagging, RF
D_8_	RF, Bagging	DT, RF	DT, KNN
D_12_	RF, DT	RF, Bagging	RF, Bagging

**Table 11:** Accuracy comparison between MLRM and meta-learning in all.

ID	Meta-Learning	MLRM	TRUE
D_1_	0.837 ± 0.005	**0.849 ± 0.008**	**0.849 ± 0.008**
D_2_	0.932 ± 0.003	**0.949 ± 0.013**	**0.949 ± 0.013**
D_3_	0.861 ± 0.002	**0.873 ± 0.003**	**0.873 ± 0.003**
D_4_	0.833 ± 0.014	**0.838 ± 0.009**	**0.838 ± 0.009**
D_5_	0.871 ± 0.030	**0.884 ± 0.030**	**0.884 ± 0.030**
D_6_	0.957 ± 0.009	**0.989 ± 0.007**	**0.989 ± 0.007**
D_7_	0.911 ± 0.009	**0.918 ± 0.010**	**0.918 ± 0.010**
D_8_	0.839 ± 0.011	**0.873 ± 0.025**	**0.873 ± 0.025**
D_9_	**0.983 ± 0.010**	**0.983 ± 0.010**	**0.983 ± 0.010**
D_10_	0.766 ± 0.026	**0.787 ± 0.009**	**0.787 ± 0.009**
D_11_	**0.995 ± 0.002**	**0.995 ± 0.002**	**0.995 ± 0.002**
D_12_	**0.912 ± 0.003**	**0.912 ± 0.003**	**0.912 ± 0.003**

**Table 12:** Accuracy comparison between MLRM and meta-learning in quinaries.

ID	Meta-Learning	MLRM	TRUE
D_1_	0.920 ± 0.002	**0.960 ± 0.027**	**0.960 ± 0.027**
D_2_	**0.927 ± 0.004**	**0.927 ± 0.004**	**0.927 ± 0.004**
D_3_	**0.894 ± 0.010**	**0.894 ± 0.010**	**0.894 ± 0.010**
D_4_	0.810 ± 0.008	**0.826 ± 0.034**	**0.826 ± 0.034**
D_6_	**0.994 ± 0.002**	**0.994 ± 0.002**	**0.994 ± 0.002**
D_7_	**0.898 ± 0.005**	**0.898 ± 0.005**	**0.898 ± 0.005**
D_8_	0.881 ± 0.003	**0.914 ± 0.004**	**0.914 ± 0.004**
D_9_	**0.968 ± 0.006**	**0.968 ± 0.006**	**0.968 ± 0.006**
D_10_	0.891 ± 0.027	**0.905 ± 0.004**	**0.905 ± 0.004**
D_11_	0.970 ± 0.009	**0.978 ± 0.005**	**0.978 ± 0.005**
D_12_	**0.853 ± 0.022**	**0.853 ± 0.022**	**0.853 ± 0.022**

**Table 13:** Accuracy comparison between MLRM and meta-learning in senaries.

ID	Meta-Learning	MLRM	TRUE
D_1_	**0.872 ± 0.006**	**0.872 ± 0.006**	**0.872 ± 0.006**
D_2_	0.893 ± 0.006	**0.927 ± 0.009**	**0.927 ± 0.009**
D_3_	**0.900 ± 0.013**	**0.900 ± 0.013**	**0.900 ± 0.013**
D_4_	0.839 ± 0.010	**0.899 ± 0.017**	**0.899 ± 0.017**
D_6_	**0.972 ± 0.012**	**0.972 ± 0.012**	**0.972 ± 0.012**
D_7_	0.899 ± 0.008	**0.903 ± 0.002**	**0.903 ± 0.002**
D_10_	**0.817 ± 0.006**	**0.817 ± 0.006**	**0.817 ± 0.006**
D_12_	0.880 ± 0.027	**0.935 ± 0.005**	**0.935 ± 0.005**

**Table 14:** Accuracy comparison between MLRM and meta-learning in septenaries.

ID	Meta-Learning	MLRM	TRUE
D_2_	**0.918 ± 0.022**	**0.918 ± 0.022**	**0.918 ± 0.022**
D_3_	**0.806 ± 0.007**	**0.806 ± 0.007**	**0.806 ± 0.007**
D_4_	0.969 ± 0.013	**0.970 ± 0.005**	**0.970 ± 0.005**
D_7_	0.964 ± 0.009	**0.968 ± 0.018**	**0.968 ± 0.018**
D_8_	0.860 ± 0.018	**0.925 ± 0.033**	**0.925 ± 0.033**
D_12_	**0.933 ± 0.022**	**0.933 ± 0.022**	**0.933 ± 0.022**

**Table 15:** Accuracy comparison between PPH and MLRM in all, quinaries, senaries, and septenaries.

ID	All		Quinaries		Senaries		Septenaries	
	MLRM	PPH	MLRM	PPH	MLRM	PPH	MLRM	PPH
D_1_	0.849 ± 0.008	**0.859 ± 0.005**	0.960 ± 0.027	**1.000 ± 0.000**	0.872 ± 0.006	**0.889 ± 0.008**	NULL	NULL
D_2_	0.949 ± 0.013	**0.959 ± 0.007**	0.927 ± 0.004	**0.958 ± 0.021**	0.927 ± 0.009	**0.955 ± 0.014**	0.918 ± 0.022	**0.941 ± 0.009**
D_3_	0.873 ± 0.003	**0.884 ± 0.011**	0.894 ± 0.010	**0.917 ± 0.013**	0.900 ± 0.013	**0.915 ± 0.009**	0.806 ± 0.007	**0.890 ± 0.010**
D_4_	0.838 ± 0.009	**0.862 ± 0.013**	0.826 ± 0.034	**0.834 ± 0.016**	0.899 ± 0.017	**0.922 ± 0.007**	0.970 ± 0.005	**0.987 ± 0.005**
D_5_	0.884 ± 0.030	**0.886 ± 0.009**	NULL	NULL	NULL	NULL	NULL	NULL
D_6_	0.989 ± 0.007	**0.997 ± 0.002**	0.994 ± 0.002	**0.999 ± 0.001**	0.972 ± 0.012	**1.000 ± 0.000**	NULL	NULL
D_7_	0.918 ± 0.010	**0.929 ± 0.004**	0.898 ± 0.005	**0.913 ± 0.005**	0.903 ± 0.002	**0.932 ± 0.013**	0.968 ± 0.018	**0.986 ± 0.006**
D_8_	0.873 ± 0.025	**0.903 ± 0.020**	0.914 ± 0.004	**0.966 ± 0.004**	NULL	NULL	0.925 ± 0.033	**1.000 ± 0.000**
D_9_	0.983 ± 0.010	**0.994 ± 0.003**	0.968 ± 0.006	**0.997 ± 0.002**	NULL	NULL	NULL	NULL
D_10_	0.787 ± 0.009	**0.797 ± 0.002**	0.905 ± 0.004	**0.921 ± 0.012**	0.817 ± 0.006	**0.882 ± 0.025**	NULL	NULL
D_11_	0.995 ± 0.002	**1.000 ± 0.000**	0.978 ± 0.005	**1.000 ± 0.000**	NULL	NULL	NULL	NULL
D_12_	0.912 ± 0.003	**0.920 ± 0.004**	0.853 ± 0.022	**0.868 ± 0.011**	0.935 ± 0.005	**0.950 ± 0.019**	0.933 ± 0.022	**0.941 ± 0.008**

## Data Availability

The data presented in this study are available upon request from the corresponding author.
